# Common thought patterns reflect generosity, fairness, and social context

**DOI:** 10.1038/s41598-026-61078-5

**Published:** 2026-07-31

**Authors:** Lisa M. Bas, Ruien Wang, Jonathan Smallwood, Anita Tusche

**Affiliations:** 1 Department of Psychology, Queen’s University, Kingston, K7L 3L3 Canada; 2 Department of Psychology, Julius Maximilians University of Würzburg, 97070 Würzburg, Germany; 3 Department of Child and Adolescent Psychiatry, Psychosomatics and Psychotherapy, University Hospital, Julius Maximilians University of Würzburg, 97080 Würzburg, Germany; 4 Centre for Neuroscience Studies, Queen’s University, Kingston, ON K7L 3L3 Canada

**Keywords:** Neuroscience, Psychology, Psychology

## Abstract

Humans generate thousands of thoughts each day, many of which concern other people. Yet it remains unclear whether these ongoing thoughts vary systematically across social contexts that demand meaningful social behavior, and whether they reflect individual differences in social preferences such as generosity and fairness. To address this question, we combined multidimensional experience sampling (MDES) with an altruism task administered online or in person (total *N* = 320). Participants made monetary sharing decisions across social contexts that varied in partner cues (distressed, neutral, unidentifiable), while intermittently reporting their ongoing thoughts using MDES. Dimensionality reduction of MDES responses using principal component analysis revealed a small set of common, interpretable, and robust thought patterns characterizing moment-to-moment cognition during social choice. Critically, the expression of these thought patterns tracked variation in social context, generosity, and model-based parameters indexing fairness preferences. Moreover, these patterns generalized to a separate non-social task that did not require social behavior, indicating that they reflect common patterns of cognition rather than task-specific artifacts. Together, these findings provide initial evidence that ongoing thought states offer reliable insights into social preferences, introducing a novel framework for studying the link between ongoing cognition and social behavior in the laboratory and everyday life.

**Supplementary Information:**

The online version contains supplementary material available at 10.1038/s41598-026-61078-5.

## Introduction

Humans are estimated to experience thousands of thoughts each day^1^, with spontaneous, internally generated cognition—such as mind wandering—occupying a substantial portion of waking life, often over 40%^2–5^. These ongoing thoughts are complex, encompassing a mixture of unconstrained cognition shaped by personal memories, goals, internal states, and external events. Importantly, a substantial proportion of spontaneous thought concerns other people and social situations^6–8^, and variation in such thought has been associated with differences in experience and behavior^9–13^. Despite growing interest in the role of spontaneous cognition in daily life, comparatively little is known about how ongoing thought patterns relate to meaningful social decisions and underlying social preferences.

Traditional accounts of social decision-making have often emphasized outcome-based preferences, such as fairness, altruism, or inequity aversion^14–18^. However, emerging work suggests that human social behavior cannot always be fully understood from economic outcomes alone. In particular, findings from behavioral economics demonstrate that changes in instructional framing and moral wording systematically relate to prosocial behavior, especially when linguistic framing highlights moral concerns^19–21^. These findings suggest that preferences may depend not only on outcomes themselves, but also on how social situations are linguistically represented and mentally judged^19^. Accordingly, researchers have argued for a broader shift from purely outcome-based to language-based utility frameworks^20^. While this perspective has primarily focused on externally provided language, internally generated cognition and language may represent an equally important—but largely overlooked—source of variation in social decision-making. Even in tightly controlled social choice tasks designed to minimize contextual influences, participants continuously generate spontaneous thoughts that may shape how social situations are interpreted and experienced. This possibility may be particularly relevant given the substantial heterogeneity often observed in social resource-allocation decisions (e.g., bimodal response distributions in dictator game contexts)^22^, suggesting that distinct forms of ongoing cognition may accompany different modes of social decision-making.

These considerations raise several important theoretical questions. What recurring forms of ongoing thought are expressed during generous and selfish behaviors? Do these thought patterns vary systematically across different social contexts and interaction partners? And are specific patterns of ongoing cognition consistently associated with individual differences in generosity and fairness-related social preferences? Addressing these questions would broaden current accounts of social decision-making by shifting attention from externally presented language and incentives toward the role of internally generated representations of social situations.

A major challenge in addressing these questions is that ongoing cognition is highly transient, multidimensional, and sensitive to both person-level and contextual factors^23^, making it difficult to capture systematically during dynamic social interactions. Multidimensional Experience Sampling (MDES) provides a promising approach to this problem. Used and validated in both controlled laboratory studies^24,25^ and daily-life contexts^26,27^, MDES involves participants linguistically rating multiple dimensions of their ongoing thoughts and experiences—including, for example, temporal focus, emotional tone, level of detail, deliberateness, and social content—thereby allowing researchers to characterize the structure of internal experiences with substantially greater precision than single-item measures of “mind wandering.” Similar to recent proposals that sentiment analysis and natural language approaches may help operationalize language-based preferences^28^, MDES provides a complementary framework for characterizing internally generated cognition during social decision-making. By applying dimensionality-reduction approaches such as principal component analysis (PCA), MDES can identify recurrent and interpretable patterns of ongoing thought that reliably emerge across individuals and settings.

Prior work demonstrates that these low-dimensional thought patterns replicate across laboratory tasks and naturalistic environments^6^, supporting their robustness and generalizability^29^. Moreover, variation in the expression of these patterns has been associated with differences in social environments (e.g., being alone vs. interacting with others), ongoing activities, task demands, and physical contexts^6,24,26,27^. For example, thought patterns characterized by episodic social cognition are more frequently reported during social interactions than during solitary contexts^26^. At the same time, several MDES-derived thought patterns have also been linked to stable individual differences, including personality traits, loneliness, well-being, emotion regulation tendencies, autism, and depression^30–34^. Together, these findings suggest that ongoing thought patterns contain both relatively stable and context-sensitive components. This combination makes MDES particularly well suited for examining social decision-making, where behavior similarly reflects both enduring social preferences (e.g., tendencies to be more or less generous across time and settings) as well as context-sensitive appraisals of interaction partners and situations that modify behaviors^14,16,35^.

Based on prior work linking spontaneous thought to social cognition^6–8,13,26^, we expected that socially oriented and emotionally reflective thought patterns would be more strongly expressed during generous choices and in socially salient contexts. This prediction is broadly consistent with findings that interventions designed to cultivate other-oriented cognition, such as compassion training and loving-kindness meditation, are associated with increases in prosocial behavior and altered responses to others’ suffering^36–39^. We further expected that thought patterns would vary systematically as a function of partner characteristics, given prior evidence of thought patterns being sensitive to features of social contexts^13,26,34^. To examine this possibility, we manipulated whether interaction partners were identifiable or unidentifiable and whether identifiable partners displayed distressed or neutral emotional expressions. The distinction between identifiable and unidentifiable partners is theoretically important because identifiable individuals are generally perceived as more socially salient and emotionally engaging than anonymous or abstract others, and identifiability has repeatedly been associated with greater prosocial responding^40–43^. Similarly, visible emotional expressions—particularly distress-related cues—may increase the salience of others’ needs and amplify socially focused cognition. We therefore expected that identifiable and emotionally expressive partners would be associated with greater expression of socially focused and emotionally elaborated thought patterns relative to unidentifiable partners. Because perceiving others’ distress elicits compassionate negative affect and increases concern for others’ versus one’s own outcome^16,37^, interactions with distressed individuals were expected to involve more other-related, less self-related, and more negatively valenced thought content. Finally, because ongoing thoughts may reflect how individuals mentally represent and evaluate social situations, we expected socially focused and emotionally elaborated thought patterns to be associated with model-based estimates of fairness preferences linked to altruistic behavior^18^.

**Fig. 1:**
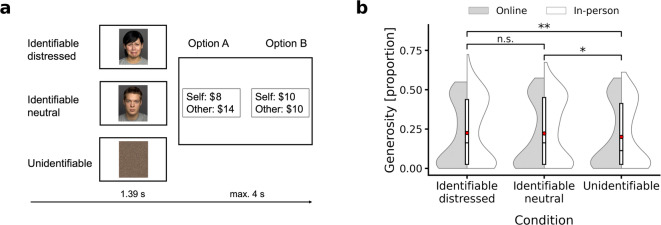
Altruism task and generosity. **(a)** On each trial, participants chose between a variable proposal (Option A) and a fixed default ($10 each; Option B), affecting payoffs for themselves (Self) and a partner (Other). The task was performed under three partner conditions (social context): identifiable distressed, identifiable neutral, and unidentifiable (control). One randomly selected trial was paid out at the end of the experiment, making choices costly to participants. Facial images (IDs 066 and 140) are reproduced from the FACES database^48^. Face models provided written informed consent for the use of their images for research purposes^48^, and online open-access publication of these images to illustrate the research methodology is permitted under the FACES Platform Release Agreement. **(b)** Split violin plots show the distribution of the participant-wise proportion of generous choices (benefiting the partner at one’s own cost) per condition (grey = online sample, white = in-person sample). Red dots mark group means, black horizontal lines mark medians, and box limits indicate interquartile ranges. Participants were more generous toward identifiable vs. unidentifiable partners (* *p* = 0.041, ** *p* = 0.019, FDR-corrected^49^, with no systematic difference between distressed and neutral identifiable partners. Overall generosity did not differ between online and in-person samples (*p* = 0.137).

To test these questions, we combined MDES with an altruism task administered to an in-person sample (*n* = 156) and an online sample (*n* = 164) yielding a total sample of 320 individuals. On each trial, participants chose between a varying monetary allocation benefiting themselves or another person and a stable default allocation yielding equal outcomes for both players (Fig. 1a). Choice behavior provided a behavioral index of generosity, operationalized as the frequency of costly prosocial choices. To characterize fairness-related social preferences associated with these decisions, we additionally estimated advantageous and disadvantageous inequity aversion using the Fehr–Schmidt computational model^18^. Across different task blocks, participants interacted with partners who were either identifiable distressed individuals, identifiable neutral individuals, or unidentifiable control partners, thereby varying the social context of decision-making. On selected trials, participants additionally reported the content of their ongoing thoughts using MDES. We then applied PCA to identify common thought patterns and used linear mixed-effects models to examine whether variation in these patterns was systematically associated with generosity, social context, and model-derived fairness preferences^24,26,34,44,45^. For completeness, we also examined whether associations between thought patterns and social behavior generalized across online and in-person testing environments. Together, this approach provides a novel framework for studying how spontaneous thought relates to social behavior across individuals, contexts, and decision environments.

## Results

### Behavior in the altruism task: generosity and fairness preferences

To examine whether ongoing thoughts are sensitive to social choice contexts and track individuals’ social preferences, we first characterized participants’ behavior in a modified dictator game (Fig. 1a). Specifically, we quantified their generosity and their fairness preferences—core considerations underlying prosocial decision-making.

***Generosity.*** We computed the proportion of generous choices each individual made in the three social contexts of the altruism task (identifiable distressed, identifiable neutral, or unidentifiable partner). Generous choices were defined as selecting the option benefiting the partner at a cost to oneself (e.g., Option A, Fig. 1a), following previous work^14–16^. The proportion of generous choices varied widely across individuals in the online (M ± SD = 0.20 ± 0.20; *n* = 164) and in-person samples (0.23 ± 0.20; *n* = 156), with bimodal distributions in both (Fig. 1b). However, generosity did not differ significantly between online and in-person participants (Wilcoxon rank sum, *p* = 0.137), so subsequent analyses combined samples (*n* = 320).

Importantly, generosity varied by social context (Friedman test, χ²(2) = 8.42, *p* = 0.015, with a small effect size, Kendall’s W = 0.013). Post-hoc tests showed lower generosity towards unidentifiable partners (0.20 ± 0.20) compared to identifiable neutral (0.22 ± 0.21; *p* = 0.041, FDR-corrected) and identifiable distressed others (0.23 ± 0.20; *p* = 0.019, FDR-corrected). Generosity did not differ between identifiable neutral and distressed conditions (*p* = 0.603, but see Supplementary Fig. S1 for visualization of individual differences). The observed increase in generosity toward identifiable others is consistent with prior work showing that identifiability and vividness (e.g., visual images of the individual, emotional facial cues) enhance prosocial behavior^46,47^.

***Fairness preferences.*** We next assessed fairness preferences—participants’ aversion to inequity in the altruism task and a powerful motivator of (pro)social behavior^50^—using a seminal model of social preference^18^. This model estimates aversion to disadvantageous inequity (*α*; being in a situation where they receive *fewer* resources than others) and advantageous inequity (*β*; being in a situation where they receive *more* resources than their interaction partners), separately for each condition of the altruism task (social context, Fig. 1a). Participants’ estimates of α and β showed a weak negative correlation (Spearman rho = − 0.19, *p* < 0.001, Supplementary Fig. S2; see Supplementary Fig. S3 for a visualization of the bimodal parameter distributions).

Advantageous inequity aversion (*β)* differed as a function of social context (χ²(2) = 11.29, *p* = 0.004, Friedman test, small effect size: Kendall’s W = 0.018). Post-hoc tests revealed stronger *β* in the identifiable distressed condition (M ± SD: 0.42 ± 0.32) compared to the unidentifiable condition (0.37 ± 0.30; *p* = 0.002, FDR-corrected). No other comparisons with the identifiable neutral condition (0.41 ± 0.32) were significant (p’s ≥ 0.103, FDR-corrected). Thus, people were more averse to self-benefiting inequity when facing visibly distressed (vs. unidentifiable) partners.

Disadvantageous inequity aversion (*α)* also varied across social contexts (χ²(2) = 86.30, *p* < 0.001, Friedman test, Kendall’s W = 0.135), being lowest with distressed identifiable partners (0.37 ± 0.18), highest with neutral identifiable partners (0.41 ± 0.15), and intermediate for unidentifiable partners (0.40 ± 0.16). All pairwise comparisons were significant (p’s < 0.001, FDR-corrected). Thus, participants were *most* concerned about being worse off than others when interacting with partners that appeared neutral and *least* concerned about it when facing a visibly distressed person.

### Common patterns of thought in altruistic choice settings and beyond

To examine ongoing thoughts in social choice contexts, participants repeatedly completed multidimensional experience sampling (MDES) during the altruism task, which required them to describe their thoughts on 14 established dimensions, each rated on a continuous rating scale (Table 1)^6,24,26,27^.

**Table 1:** Overview of multidimensional experience sampling (MDES) questions used to characterize ongoing thoughts.

Dimension	Statement	Scale low	Scale high
Task	My thoughts were focused on the task I was performing:	Not at all	Completely
Future	My thoughts involved future events:	Not at all	Completely
Past	My thoughts involved past events:	Not at all	Completely
Emotion	The emotion of my thoughts was:	Negative	Positive
Modality	My thoughts were in the form of:	Images	Words
Detail	My thoughts were detailed and specific:	Not at all	Completely
Deliberate	My thoughts were:	Spontaneous	Deliberate
Problem	I was thinking about solutions to problems (or goals):	Not at all	Completely
Diverse	My thoughts were:	One topic	Many topics
Intrusive	My thoughts were intrusive:	Not at all	Completely
Memory	My thoughts were linked to information from:	Environment	Memory
Self	My thoughts involved myself:	Not at all	Completely
OtherClose	My thoughts involved other people close to me:	Not at all	Completely
OtherDistant	My thoughts involved other people NOT close to me:	Not at all	Completely

Note: Rated on a continuous 100-point visual analog scale. In visualizations, the *Modality* dimension is labeled as *Words* in word clouds to enhance interpretability by using terminology more intuitively understood.

**Fig. 2:**
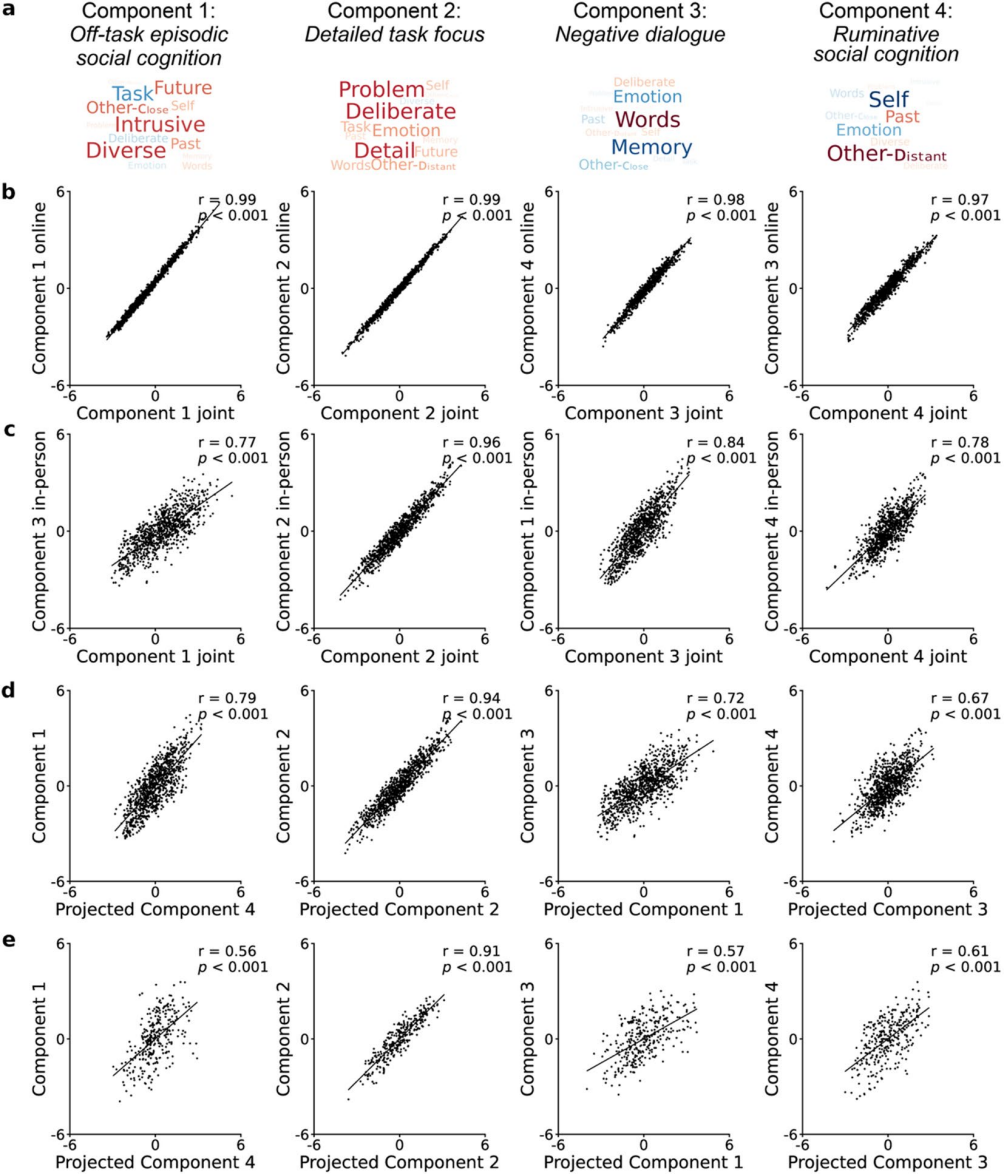
Reliability and generalizability of common thought patterns. **a.** Word clouds illustrate the four principal components (common thought patterns) derived from MDES data in the altruism task, explaining 52% of variance in ongoing cognition (PCA on the joint sample of 320 participants; 1,920 observations). Word size reflects the magnitude of each MDES item’s loading (see Table 2), and color indicates direction (red = positive; blue = negative). **b–c.** Components extracted separately in the online and in-person samples (y-axes) closely matched those obtained from the joint sample (x-axes), demonstrating robustness across samples and task environments (note that component ordering may differ between the joint and sample-specific PCAs). **d**. Component loadings from the in-person sample projected successfully onto the online sample, supporting cross-sample (environment) generalizability. **e.** Component loadings from the altruism task also projected onto MDES data from a separate free-viewing session approximately seven days later (*n* = 156), indicating that these thought patterns extend beyond altruistic decision-making to contexts without explicit social demands or specific social behaviors.

***Common thought patterns.*** To identify common patterns of ongoing thought, a principal component analysis (PCA) with Varimax rotation was applied to all MDES observations (*n* = 1,920 across samples), following previous studies^6,24,26,27,51^. Bartlett’s test of sphericity was significant (χ²(91) = 3895.52, *p* < 0.001), and the Kaiser-Meyer-Olkin measure indicated good sampling adequacy (KMO = 0.73), confirming the suitability of PCA. Four components with eigenvalues greater than 1 emerged, explaining 52% of the variance in the MDES data (Supplementary Fig. S4). PCA loadings (Table 2) from the four components were used to generate thought word clouds to visualize common patterns of ongoing cognition in social choice settings (Fig. 2a). We named the four components based on the MDES dimensions that dominated their composition and based on labels used in prior work to characterize common thought patterns.

Component 1 (19% of the variance) was characterized by a strong negative loading on task and high positive loadings on past, close others, self, and diverse. We labeled this component “off-task episodic social cognition,” as it reflects broad episodic and social thoughts about oneself and close others that were unrelated to the task. Component 2 (15% of the variance) was labeled ‘detailed task focus’ due to high positive loadings on items detailed, deliberate, problem, and task, capturing concentrated and goal-directed thoughts about the task at hand. Component 3 (9% of the variance) was marked by positive loadings on words and negative loadings on emotion and memory, indicating verbal, negatively valenced thoughts tied to the immediate environment; we labeled this pattern “negative dialogue.” Component 4 (9% of the variance) was distinguished by high loadings on past and distant others, combined with negative loadings on emotion and self. We labeled this pattern “ruminative social cognition,” as it reflects negative, past-oriented thoughts, and focuses on distant others (unrelated to oneself).

The first three components replicate common thought patterns that have previously been observed in both laboratory and real-world contexts^23,26,27,52^, highlighting their robustness and generalizability. The fourth component, however, appears novel and may reflect cognitive processes more specific to altruistic choice settings. Hereafter, we will use these component labels to discuss the link between common thought patterns and social contexts, generosity, and fairness preferences. However, we acknowledge that other labels could be used for the four components. Consistent with prior MDES research^26,27,33,44,53^, the identification of common thought patterns was approached in a largely data-driven manner to accommodate the substantial heterogeneity of ongoing experience^23^. Accordingly, PCA was used to characterize low-dimensional patterns emerging from the multidimensional thought reports rather than to test highly specific hypotheses regarding the precise component structure.

**Table 2:** Thought data loadings generated by PCA with Varimax rotation.

MDES Item	Component 1	Component 2	Component 3	Component 4
Task	−0.37	−0.24	−0.07	−0.04
Future	0.37	−0.18	0.01	0.01
Past	0.32	−0.10	−0.17	−0.32
Emotion	−0.10	−0.33	−0.34	0.32
Modality	0.06	−0.13	0.65	0.09
Detail	0.01	−0.47	−0.03	0.00
Deliberate	−0.17	−0.47	0.19	−0.12
Problem	0.08	−0.47	−0.02	−0.06
Diverse	0.46	0.13	0.03	−0.08
Intrusive	0.43	0.04	0.09	0.06
Memory	0.12	−0.13	−0.55	0.02
Self	0.16	−0.17	0.18	0.59
OtherClose	0.37	−0.05	−0.22	0.14
OtherDistant	0.09	−0.22	0.08	−0.62

Note: We refer to components 1 to 4 as common thought patterns in the main text; component 1 = ‘off-task episodic social cognition,’ component 2 = ‘detailed task focus,’ component 3 = ‘negative dialogue,’ and component 4 = ‘ruminative social cognition.’

*Stability*,* reliability*,* and generalizability of thought patterns.* We ran several supplemental analyses to make sure that the thought patterns we identified from the MDES data were reliable, stable, and could generalize across different participants and task settings. First, a bootstrapped split-half reliability analysis was conducted to confirm that the four-component solution (Fig. 2a) provided a reasonable description of our data on ongoing thoughts. This analysis repeatedly divided the MDES data into two random samples and checked whether the patterns in one half matched those in the other. The strong homologue similarity score (Pearson’s *r* = 0.96, 95% CI [0.93, 1.00]) indicates that our four-component solution is highly reproducible.

Second, following previous methodology^24,54^, we quantified reliability using intraclass correlations (ICCs) for each thought pattern across all task conditions and samples. Supplementary Table S1 shows the mean ICC measures for each task condition, component, and sub-sample, indicating good reliability for all four components (ICC range = 0.68–0.89).

Third, we checked whether the thought patterns were present in both online and in-person samples (for the main PCA, we collapsed across all participants and observations). We ran separate PCAs on sample-specific MDES data (Supplementary Fig. S4) and compared the component scores to those from the combined analysis^26,55^. The identified components were strongly correlated across samples (Pearson’s r’s = 0.76–0.99, all p’s < 0.001), suggesting their presence regardless of the sample-specific task environments (in-person, online; Fig. 2b-c).

Fourth, as a more direct test of the generalizability of thought patterns across samples and choice environments, we projected the component loadings identified in one sample (in-person) onto the data of the other sample (online; see Methods and previous implementations^29,44^). The projected patterns matched well, indicating that our thought patterns generalize across samples and task environments (Pearson’s r’s = 0.67–0.94, all p’s < 0.001; Fig. 2d), to a comparable level as in prior work^29,44^.

Finally, we tested whether the thought patterns identified in the altruism task also appear in contexts unrelated to social decision-making. Participants completed a separate computerized free viewing task (see Methods), in which they observed complex real-world scenes without any meaningful social or decision-making demands, on average seven days after the altruism task. We projected the thought patterns derived from the MDES data in the altruism task onto the MDES data obtained in the free viewing task (Supplementary Fig. S4). We found significant correlations between the patterns observed in both tasks (Pearson’s r’s = 0.56–0.91, all p’s < 0.001; Fig. 2e). This finding indicates that the identified thought patterns generalize—at least to some degree—beyond social choice situations to settings where people freely view complex everyday scenes without any demands on social behavior.

Taken together, these analyses demonstrate that the PCA captured common patterns of ongoing thought across different samples and task environments (online vs. in-person), and even contexts unrelated to social decision-making (free viewing task).

### Association of generosity, social context, and task environment with ongoing thought patterns

**Fig. 3:**
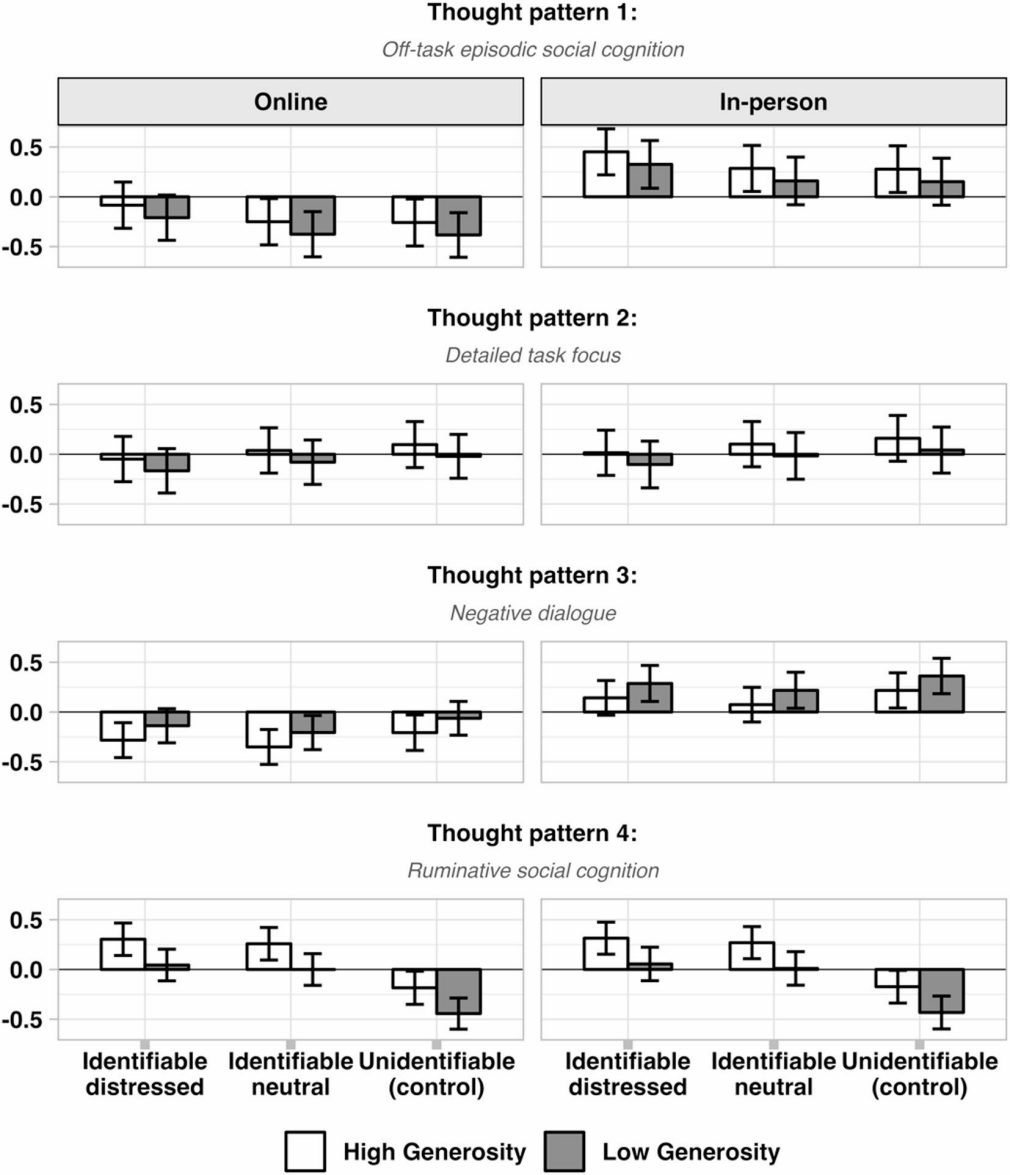
Common thought patterns vary as a function of social and environmental factors. Predicted prevalence of the four thought patterns (rows) from four linear mixed models is shown across *(i)* high vs. low generosity groups (white vs. grey bars), *(ii)* social context (interaction partner: identifiable distressed, identifiable neutral, or unidentifiable; y-axis), and *(iii)* task environment (online vs. in-person; left vs. right panels). Bars represent predicted means for each experimental cell, with error bars showing 95% confidence intervals.

Having established the reliability and generalizability of common patterns of ongoing thought, we next examined whether they are systematically associated with social and contextual factors. Specifically, we tested whether ongoing thought patterns varied as a function of participants’ generosity, the characteristics of their interaction partner, and the task environment. Four linear mixed models were conducted, each predicting one of the thought components as the dependent variable, following prior research^26,27^. The models included three key predictor variables: participants’ generosity level (low vs. high, binarized due to the observed bimodal distribution; Fig. 1b), partner characteristics in the altruism task (social context: identifiable distressed, identifiable neutral, or unidentifiable others), and task environment (online vs. in-person). Task environment was included to control for and evaluate potential environmental influences in thought expression, which, to our knowledge, have never been systematically examined for in-person vs. online settings of the same task. Age was included as a covariate, and random intercepts for participants accounted for repeated measurements. Interaction terms were not included because formal model comparisons favored simpler models (Supplementary Table S2). Estimates reported in Table 3 are unstandardized, representing the deviation of each factor level from the grand mean of all conditions (sum coded categorical predictor variables, see Methods). Table 3 also indicates results that remain significant after Bonferroni correction for multiple comparisons across the four models. In the main text below, we report results for the effects of interest (for a complete list, see Table 3), including Bonferroni-corrected p-values across all four models and uncorrected p-values for each individual model. A visualization of the effects is provided in Fig. 3.

***Model 1: Off-task episodic social cognition.*** We observed a significant main effect of task environment (b = −0.27, 95% CI [−0.43, −0.11], t(316) = −3.29, p_uncor_ = 0.001, p_Bonferroni_ = 0.004), with participants in the online sample reporting fewer off-task episodic social cognition thoughts relative to the grand mean, and those in-person reporting more. Social context was also significantly associated with this thought pattern: off-task episodic cognition was more prevalent when interacting with an identifiable distressed partner relative to the grand mean (b = 0.11, 95% CI [0.05, 0.17], t(1597) = 3.64, p_uncor_ < 0.001, p_Bonferroni_ = 0.001), but did not significantly differ for identifiable neutral partners (b = −0.05, 95% CI [−0.11, 0.01], t(1596) = −1.70, p_uncor_ = 0.089, p_Bonferroni_ = 0.354). Generosity level was not significantly associated with off-task episodic thoughts (b = 0.06, 95% CI [−0.02, 0.14], t(1743) = 1.52, p_uncor_ = 0.128, p_Bonferroni_ = 0.512). Instead, there was a weak but significant effect of age, with lower expressions of off-task episodic social cognition in older individuals (b = −0.03, 95% CI [−0.05, −0.01], t(1596) = −3.38, p_uncor_ < 0.001, p_Bonferroni_ < 0.003).

***Model 2: Detailed task focus.*** We observed a significant main effect of social context, such that thoughts characterized by detailed task focus were less prevalent when participants interacted with an identifiable distressed partner (b = −0.08, 95% CI [−0.13, −0.02], t(1599) = −2.70, p_uncor_ = 0.007, p_Bonferroni_ = 0.028). No other significant effects were observed for this thought pattern (all p_uncor_ > 0.127).

***Model 3: Negative dialogue.*** Task environment was significantly associated with thoughts characterized by negative dialogue (b = −0.21, 95% CI [−0.33, −0.09], t(317) = −3.47, p_uncor_ = 0.001, p_Bonferroni_ = 0.002), with a lower prevalence of this thought pattern in the online sample and more in-person compared to the grand mean. Social context also had an effect: negative dialogue decreased when interacting with identifiable neutral partners (b = −0.07, 95% CI [−0.12, −0.02], t(1598) = −2.97, p_uncor_ = 0.003, p_Bonferroni_ = 0.012). At a more lenient threshold (uncorrected), generosity was associated with negative dialogue: participants in the high generosity group reported fewer negative dialogue thoughts relative to the grand mean, while those in the low generosity group reported more. However, this effect did not survive Bonferroni correction across the four models (b = −0.07, 95% CI [−0.13, −0.01], t(1726) = −2.30, p_uncor_ = 0.022, p_Bonferroni_ = 0.086), so we did not pursue follow-up analyses on this link between generosity and internal negative dialogue.

***Model 4: Ruminative social cognition.*** Social context strongly affected this thought pattern: ruminative social cognition were more prevalent when interacting with identifiable distressed (b = 0.18, 95% CI [0.13, 0.23], t(1596) = 7.05, p_uncor_ < 0.001, p_Bonferroni_ < 0.001) or identifiable neutral partners (b = 0.13, 95% CI [0.08, 0.18], t(1595) = 5.26, p_uncor_ < 0.001, p_Bonferroni_ < 0.001) relative to the grand mean. Generosity also was significantly associated with the occurrence of this type of thought: more generous participants reported higher levels of ruminative social cognition relative to the grand mean (b = 0.13, 95% CI [0.07, 0.19], t(1508) = 4.10, p_uncor_ < 0.001, p_Bonferroni_ < 0.001), and significantly fewer thoughts of this type in less generous participants.

Overall, these findings indicate that common patterns of ongoing thought systematically vary as a function of generosity, social context (partner characteristics) and task environment (online vs. in-person).

We conducted three sets of supplemental analyses to assess the robustness and temporal structure of these effects. First, re-estimating the models using the continuous number of generous choices—rather than a binarized high vs. low generosity grouping used to accommodate the binary distribution of observed generosity—confirmed the qualitative pattern of results across all four models (Supplementary Table S3). Second, we tested the reverse association (using generosity as the dependent variable and common thought patterns as predictors) and found that common thought patterns were related to individual differences in generosity (see Supplementary Tables S4, S5, and S6; for supplemental models predicting social context and task environment from thought patterns, see note of Supplementary Table S4). Third, to further examine the temporal relationship between ongoing thought patterns and social behavior (generosity), we conducted two sets of time-lagged analyses. These models test whether variation in one variable tends to precede variation in another, thereby providing stronger evidence regarding temporal ordering. We first asked whether the expression of thought pattern 4 (ruminative social cognition), which showed the most robust link with generosity, predicted the generosity level on the subsequent trial. Ruminative social cognition was positively associated with increased generosity on trials immediately *following* the MDES thought probes (b = 0.18, 95% CI [0.01, 0.35], OR = 1.20, 95% CI [1.01, 1.41], z = 2.11, p = 0.03; see Supplementary Table S7 for full results). We next examined the reverse temporal relationship and found that greater generosity on the trial immediately *preceding* the MDES probe was associated with increased expression of ruminative social cognition (b = 0.13, 95% CI [0.01, 0.25], t(1308) = 2.17, p = 0.03; see Supplementary Table S8 for full details). Together, these supplemental time-lagged analyses suggest a bidirectional relationship between ruminative social cognition and generosity, consistent with a dynamic interplay between ongoing cognition and social behavior.

**Table 3:** Common thought patterns vary as a function of generosity, social context, and task environment.

Predictors	Thought Pattern 1 (off-task episodic social cognition)				Thought Pattern 2 (detailed task focus)				Thought Pattern 3 (negative dialogue)				Thought Pattern 4 (ruminative social cognition)			
	Estimate	CI	Statistic	*p*	Estimate	CI	Statistic	*p*	Estimate	CI	Statistic	*p*	Estimate	CI	Statistic	*p*
(Intercept)	0.92	[0.38, 1.46]	3.32	**0.001** ^**‡**^	−0.19	[−0.73, 0.35]	−0.7	0.485	0.05	[−0.36, 0.46]	0.22	0.823	0.04	[−0.33, 0.41]	0.19	0.851
Environment (Online)	−0.27	[−0.43, −0.11]	−3.29	**0.001** ^**‡**^	−0.03	[−0.19, 0.13]	−0.39	0.694	−0.21	[−0.33, −0.09]	−3.47	**0.001** ^**‡**^	−0.01	[−0.11, 0.10]	−0.11	0.914
Generosity (High)	0.06	[−0.02, 0.14]	1.52	0.13	0.06	[−0.02, 0.13]	1.52	0.127	−0.07	[−0.13, −0.01]	−2.30	0.022	0.13	[0.07, 0.19]	4.10	**< 0.001** ^**‡**^
Context (Identifiable distressed)	0.11	[0.05, 0.17]	3.64	**< 0.001** ^**‡**^	−0.08	[−0.13, −0.02]	−2.70	**0.007** ^**‡**^	0	[−0.05, 0.04]	−0.07	0.941	0.18	[0.13, 0.23]	7.05	**< 0.001** ^**‡**^
Context (Identifiable neutral)	−0.05	[−0.11, 0.01]	−1.70	0.087	0.01	[−0.05, 0.07]	0.32	0.747	−0.07	[−0.12, −0.02]	−2.97	**0.003** ^**‡**^	0.13	[0.08, 0.18]	5.26	**< 0.001** ^**‡**^
Age	−0.03	[−0.05, −0.01]	−3.38	**0.001** ^**‡**^	0.01	[−0.01, 0.03]	0.72	0.469	0	[0.02, 0.01]	−0.21	0.834	0	[−0.01, 0.01]	−0.18	0.855

Note: ^‡^ indicates p-values that survive Bonferroni correction for multiple comparisons across the four independent models.

### Thought patterns reflect individuals’ fairness preferences associated with differences in (pro)social behaviors

So far, we have shown that the expression of four common patterns of ongoing thought is sensitive to generosity, social and environmental factors. For example, variance in people’s levels of generosity was associated with a thought pattern characterized by ruminative social cognition (thought pattern 4; Table 3). Building on this result, we next asked whether this thought pattern is also associated with fairness considerations that relate to differences in generosity across individuals and contexts.

To address this question, we used a well-established model of social preferences^18^ to characterize the impact of fairness preferences on altruistic choices. The model posits that variance in prosocial behavior can be explained by differences in aversion to inequity when participants are better off than others (advantageous inequity) or worse off than others (disadvantageous inequity). Using model-based estimates of participants’ advantageous inequity aversion (β) and disadvantageous inequity aversion (α), we examined whether these fairness preferences account for individual differences in ruminative social cognition (thought pattern 4), previously linked with generosity.

To this end, we conducted a linear mixed model predicting thought pattern 4 (dependent variable) from *α* and *β* (predictors). Due to the bimodal distribution of estimated parameters, α and β were treated as binary variables (Supplementary Fig. S3). For consistency with our main models, environment (online vs. in-person), social context (partner characteristics), age, and a random intercept for participants were also included as predictors.

We observed a significant main effect of advantageous inequity aversion (*β*) on ruminative social cognition (b = 0.11, 95% CI [0.05, 0.17], t(1587) = 3.57, p < 0.001; Table 4). Participants with high *β*—more averse to being in a situation where they receive more resources than others—reported greater ruminative social cognition relative to the grand mean, whereas those with low *β* were less prone to express this thought pattern. This suggests that individual differences in advantageous inequity aversion may contribute to the link between altruism and ruminative social thoughts about distant others. We observed a non-significant main effect of disadvantageous inequity aversion *α* (b = −0.02, 95% CI [−0.07, 0.02], t(1587) = −1.03, p = 0.302. Consistent with our previous results, social context also had a significant impact: participants exhibited higher levels of ruminative social cognition when interacting with an identifiable distressed partner (b = 0.17, 95% CI [0.12, 0.22], t(1599) = 6.90, p < 0.001) or an identifiable neutral partner (b = 0.13, 95% CI [0.08, 0.18], t(1594) = 5.30, p < 0.001; Table 4).

In sum, we found that the expression of a common pattern of ongoing thought in social choice settings—ruminative social cognition—was associated with differences in participants’ fairness preferences, specifically advantageous inequity aversion (β), that guide prosocial decision-making. Supplemental analyses using continuous estimates of α and β confirmed these results (Supplementary Table S9). These findings establish a novel link between ongoing cognition and key social considerations underlying social decision-making.

**Table 4:** Thought pattern 4 (ruminative social cognition) is associated with fairness preferences.

Predictors	Estimates	CI	Statistic	*p*
(Intercept)	0.04	[−0.33, 0.41]	0.22	0.825
Advantageous inequity aversion (high *β*)	0.11	[0.05, 0.17]	3.57	< 0.001
Disadvantageous inequity aversion (high *α*)	−0.02	[−0.07, 0.02]	−1.03	0.302
Environment (online)	−0.01	[−0.12, 0.10]	−0.15	0.877
Context (identifiable distressed)	0.17	[0.12, 0.22]	6.90	< 0.001
Context (identifiable neutral)	0.13	[0.08, 0.18]	5.30	< 0.001
Age	−0.00	[−0.02, 0.01]	−0.23	0.821

Note: CI = confidence interval.

## Discussion

Humans spend a large portion of their waking lives immersed in ongoing thoughts, many of which concern other people. Yet it remains largely unknown how these spontaneous mental experiences relate to the social choices we make and the social preferences underlying them. Do the fleeting thoughts we have in the moments surrounding a decision reveal something fundamental about our preferences for fairness, generosity, or concern for others? Our study provides initial evidence that they do. By integrating multidimensional experience sampling (MDES) with an established social decision-making paradigm, we identified robust and interpretable patterns of ongoing thought that emerged consistently across participants, task environments, and even in a non-social task context. Importantly, these thought patterns tracked features of the social choice context as well as individual differences in social preferences. For example, ruminative social cognition—marked by a focus on ongoing thoughts on distant others and past events—was heightened in individuals who were more generous and more averse to advantageous inequity. Moreover, thought patterns were sensitive to social contexts; interacting with identifiable partners, especially distressed ones, increased both off-task episodic social cognition and ruminative social cognition. Together, these findings suggest that ongoing thoughts provide meaningful insight into the cognitive states that are linked to human social behavior. By linking moment-to-moment cognition with prosocial decision-making, our work introduces a novel framework for understanding how the private world of thoughts relates to the public world of social action.

Ruminative social cognition (thought pattern 4) showed the clearest association with prosocial behaviors (generosity). This common thought pattern is distinguished by a dissociation between self- and other-focused features of cognition, mirroring core features of formal models of altruistic choice that describe prosocial behavior as a value-based trade-off between self-regard and other-regard^16,17,56,57^. These choice-relevant attributes have been shown to reliably guide social decisions^18,58–61^, with lower weighting of self-regard and higher weighting of other-regard yielding greater prosociality^62–65^. Extending this value-based framework of social decision-making, we demonstrate that the content of unconstrained cognition follows similar principles: thinking less about oneself and more about (distant) others was associated with increased generosity. To our knowledge, this is the first evidence that features of spontaneous thought track stable variance in social preferences across people and contexts. In this sense, ongoing thoughts may reliably index both the likelihood of acting prosocially and the contexts under which they emerge, evidenced by an increased occurrence of this other-oriented thought pattern.

The context-sensitivity of ruminative social cognition and other thought patterns—including off-task episodic social cognition and detailed task focus (thought patterns 1 and 2)—aligns with prior evidence that ongoing cognition varies meaningfully with situational demands^24,26,27,34^. For instance, episodic social cognition has previously been shown to increase during real-world social interactions compared to situations in which participants were alone or with others but not interacting^27^, with amplified effects during COVID-19 lockdowns when interactions were less frequent but more socially significant^26^. Our findings extend this thought–context mapping to specific features of social interactions, such as partner identifiability and emotional state. This extension is particularly timely given that increasing portions of modern social interaction occur online or with artificial agents, where identifiability can be markedly reduced. Crucially, context-driven shifts in unconstrained cognition were associated with behavioral changes: participants behaved more generously toward identifiable individuals, especially those displaying distress. Despite sensitivity to these contextual cues, the relationship between ruminative social cognition and generosity remained stable across online and in-person environments, underscoring the robustness of the effect and supporting the use of online testing for future research to further probe the mapping of ongoing thoughts and social behaviors.

Well-supported economic models of social preferences distinguish between different considerations that explain variance in observed generosity (e.g., perceived unfairness or inequity aversion^18,58,66,67^). Ruminative social cognition was not just linked to participants’ generosity but also inequity aversion. Participants with stronger advantageous inequity aversion—those uncomfortable with being better off than others—experienced more ruminative social cognition. One interpretation is that thinking about others or past events may evoke discomfort or guilt when one benefits disproportionately, increasing the willingness to share resources in subsequent trials, even at a cost to oneself. We did not find a clear association between disadvantageous inequity aversion and any thought pattern, possibly because the randomized presentation of trial types within blocks may have blurred aversion-specific cognitive signatures. Future work using MDES probes targeted separately at advantageous versus disadvantageous contexts (e.g., in different blocks) may clarify this relationship. More broadly, exploring links to other key social preference-related processes—such as reciprocity^68,69^, norm compliance^70^, reputation management^71,72^—and investigating the neural substrates of the thought–generosity mapping^73^ represent compelling directions for future research.

Overall, our findings suggest that MDES-derived thought patterns are unlikely to reflect solely stable preferences or purely context-driven fluctuations. Instead, the results are most consistent with a hybrid account in which thought patterns contain both trait-like and state-like components that show dynamic temporal associations with behavior. First, strong reliability, cross-sample reproducibility, and generalization across task environments and a free-viewing paradigm point to a stable component reflecting enduring individual differences in ongoing cognition. Second, systematic variation across experimentally manipulated social contexts (e.g., partner identifiability and emotional expression) supports a context-sensitive state component linked to situational demands. Third, bidirectional lagged associations between thought patterns and generosity suggest that cognition and behavior are temporally related over time. Together, these findings indicate that MDES-derived thought patterns reflect a mixture of stable dispositions, context-sensitive fluctuations, and ongoing cognition–behavior dynamics. More broadly, this interpretation aligns with contemporary views in psychology that many constructs combine stable between-person differences with dynamic within-person variation, with their relative contributions varying across contexts and timescales. Similar hybrid frameworks have been proposed for constructs such as mind-wandering, affect, self-control, and rumination^74–77^.

Although our results provide new insights into the functional coupling between ongoing cognition and social preferences, several limitations warrant consideration. First, our data speak to the generalizability of the identified thought patterns and generosity across online and in-person laboratory environments, but not to real-world social contexts. Prior work indicates that certain features of mind-wandering—such as social and emotional content—are preserved outside the lab^78^, and recent evidence shows that MDES-based thought patterns are sensitive to daily life contexts^26,27,34^. Thus, we speculate that similar thought–behavior relationships may arise in more naturalistic settings, though this remains to be tested. Second, our findings are specific to generosity in an economic game framework. Future research should evaluate whether our approach generalizes to other forms of prosocial behavior^79,80^, including cooperation or altruistic punishment to enforce social norms. Finally, the causality of the thought–behavior relationship remains unresolved. While our supplemental time-lagged analyses suggest a bidirectional relationship in the temporal ordering of the two variables (ruminative social cognition and generosity), the design does not allow us to determine definitively whether thoughts influence behavior, behavior influences thoughts, or both. Experimental paradigms optimized to test causal dynamics will be essential to advancing this line of research.

In summary, our findings reveal that common patterns of moment-to-moment thoughts are systematically linked to social context, meaningful (pro)social behavior, and social preferences, highlighting ongoing cognition as a promising new lens for understanding human social decision-making in in-person and online environments.

## Methods

### Participants

We collected data from two independent samples in different task environments: an online sample of 166 participants recruited through Prolific (http://www.prolific.co) and an in-person sample of 157 participants recruited from the local community via flyers and online advertisements. Eligibility criteria included fluency in English, normal vision, and no current or previous history of psychological disorders. One outlier in the in-person sample—identified using the *isoutlier* function in MATLAB2021b and exceeding three standard deviations from the mean generosity—was excluded, resulting in a final in-person sample of 156 participants (102 female; M ± SD: 22 ± 5.60 years). Two online participants were excluded for not meeting all eligibility criteria, yielding a final online sample of 164 participants (78 female; M ± SD: 31 ± 7.79 years).

Online participants completed a single session that included the altruism task. In-person participants attended two lab sessions on separate days, completing either the altruism task or a free-viewing task that did not involve meaningful social actions or prosocial decision-making. The sessions were separated by an average of 7 days (range: 1–23 days), and task order was randomized across participants. Participants were compensated at £7/hour (online) or $10/hour (in-person). Both samples received an additional payment based on one randomly selected trial from the altruism task to ensure incentive-compatible behavior, aligning observed social behaviors with actual preferences. The study was approved by the Queen’s University Health Sciences and Affiliated Teaching Hospitals Research Ethics Board (HSREB). All procedures were conducted in accordance with relevant guidelines and regulations, and all participants provided written informed consent.

### Behavioral tasks

***Altruism task.*** To assess individual differences in generosity and fairness preferences, participants in both samples completed a modified version of the widely used dictator game^81^ (Fig. 1a). In each trial, participants chose between two monetary offers via key press. One offer (Option A) varied across trials, with different allocations to themselves ($Self) or another player ($Other; $0–$20). The other offer (Option B) was a constant default ($10 for each player). Offers were presented on the left and right sides of the screen, with the positions of the varying and default offers randomized across trials. For each participant and task block, 80% of trials (i.e., 40 out of 50) required a trade-off between payoffs to self and other. In these critical trials, the varying offer could either benefit the participant at a cost to the other ($Self > $Other) or benefit the other at a cost to the participant ($Other > $Self). The remaining 20% of trials (i.e., 10 per block) did not require a trade-off relative to the default allocation; the varying option either increased both players’ payoffs ($Self > $10 and $Other > $10) or did not increase either player’s payoff ($Self < $10 and $Other < $10). Consistent with prior approaches, participants’ generosity was quantified based on trials requiring trade-offs, with a choice defined as generous when participants selected the option that benefited the other at a cost to themselves^14–16^.

To examine the influence of social context^82^, participants completed the altruism task under three partner conditions (separate blocks): ‘identifiable distressed,’ ‘identifiable neutral,’ and ‘unidentifiable’ (control). In the ‘identifiable distressed’ blocks, participants viewed a brief video (1.39 s) of a dynamic sad face prior to each monetary offer. In the ‘identifiable neutral’ blocks, a neutral face video (1.39 s) was presented, and in the control blocks, participants saw a scrambled face image (1.39 s). All face stimuli were drawn from the FACES database^48,83^, and participants were presented with a cover story framing the other players as previous participants who had agreed to have their facial expressions recorded and permitted the use of these for research purposes^48^.

Participants completed 300 trials in total (in six blocks, two blocks per partner condition, 50 trials per block, yielding 100 trials per condition, with 80 trials of interest that required a trade-off between payoffs to self and other per conditions). Block order was pseudorandomized across participants. The number of observed generous choices per block allowed us to examine the link between generosity and ongoing cognition, as well as how altruism varied with contextual social information (i.e., partner characteristics). These choices were also used in a computational model to estimate individual fairness preferences (advantageous and disadvantageous inequity aversion), an important factor explaining individual differences in (pro)social behavior.

***Multidimensional experience sampling (MDES).*** Ongoing thoughts during the altruism task (and the free viewing task, see below) were assessed using multidimensional experience sampling (MDES)^84^. Participants were probed six times during the altruism task (twice per condition, once in each block), reporting the content of their thoughts via 14 statements (Table 1). Statement order was randomized except for the first item, “My thoughts were focused on the task I was performing.” Ratings were given on a continuous scale from 1 to 100, with a random starting point, and later rescaled by dividing by 10 to align with typical 1–10 scales used in previous studies^24,52^. MDES data were used to identify common thought patterns (see below).

***Free viewing task and MDES.*** To assess the generalizability of thought patterns observed in the altruism task, in-person participants (*n* = 156) completed a free viewing task on a separate day^85,86^. The average delay between sessions was seven days, minimizing cross-session dependencies. Participants viewed 300 naturalistic images from the OSIE dataset^86^, depicting complex real-world indoor and outdoor scenes, and were instructed to “View these images as you naturally would.” Images were displayed for 3 s in four blocks of 75 images each, separated by a fixation cross for 0.5, 1, or 1.5 s (random jitter). Breaks of 1–5 min between blocks reduced fatigue. Block order was counterbalanced across participants (two sequences: 1–4 or 4–1), while image order within each block was fixed. MDES probes (Table 1) were administered twice per participant (after the second and fourth blocks, independent of the block order sequence). Eye-tracking data were also collected for this task, but are reported elsewhere.

### Analysis

***Generosity (altruism task).*** We assessed generosity in the altruism task on the trials of interest (80%) that required a trade-off between participants’ own payoff ($Self) and that of their partner ($Other). Following prior work^14,15,62^, choices were classified as generous if participants *(i)* accepted a proposal that benefited the partner at a personal cost ($Self < $Other) or *(ii)* rejected a proposal that benefited themselves at the partner’s expense ($Self > $Other), thereby reverting to the stable default option. The fraction of generous choices (range 0–1) was estimated for each participant, providing a model-free measure of generosity in each condition-specific context (partner characteristics: identifiable distressed, identifiable neutral, unidentifiable control; Fig. 1a) and task environment (online, in-person). One participant in the in-person sample was excluded as an outlier (three SDs from the mean; identified with the *isoutlier* function in Matlab2021b).

We first compared overall generosity between online and in-person samples. As the data were non-normally distributed (Fig. 1b), we used a Wilcoxon rank sum test to assess group differences in R^87^. Second, we examined if social context was associated with differences in people’s generosity, operationalized through partner characteristics. Given comparable generosity levels across task environments, this analysis was conducted on the joint sample. We used the Friedman test in the R *stats* package to analyze condition-specific fractions of generous choices^87^. We conducted post-hoc, non-parametric pairwise comparison tests using the *PMCMRplus* package in R, with the FDR-corrected Conover test^88^. Third, generosity levels also served as predictor variables in four mixed linear models linking generosity (and social context and task environment) to common patterns of ongoing thought (see below). Given the bimodal distribution of observed generosity scores (Fig. 1b), the primary mixed linear models used a binarized variable of high vs. low generosity (median split); supplemental analyses with continuous generosity scores confirmed the robustness of our results. All p-values in this manuscript are reported two-tailed.

***Fairness preferences (altruism task).*** To characterize participants’ fairness preferences, we applied the established Fehr-Schmidt inequity aversion model to the choice data obtained during the altruism task^18^. This model is widely used to quantify how fairness considerations guide social decision-making and prosocial behaviors^89^. To obtain participant-specific parameters for advantageous and disadvantageous inequity aversion, subjective utility was modeled as follows:

1 $$\:U\:=\:\$Self - \alpha\:\cdot max(\$Other-\$Self,0)- \beta\: \cdot max(\$Self-\$Other,0)$$

where utility (*U)* depends on the participant’s own payoff ($Self) and the payoff allocated to the other person ($Other). The parameter *α* captures disadvantageous inequity aversion (i.e., aversion to receiving less than the other person), whereas *β* captures advantageous inequity aversion (i.e., aversion to receiving more than the other person)^18^.

Consistent with the standard formulation of the Fehr–Schmidt model, *β* was constrained to the interval ([0,1]), and *α* was constrained such that α ∈ [β,5]. The lower-bound constraint (α ≥ *β*) reflects the theoretical assumption that disadvantageous inequity is generally experienced as more aversive than advantageous inequity. The upper bound imposed on α was included solely to improve numerical stability during estimation and does not constitute a theoretical restriction of the model.

Trial-wise utilities were transformed into binary choice probabilities using a softmax observation model with an inverse-temperature parameter capturing stochasticity in participants’ choices. Model parameters were estimated separately for each participant using variational Bayesian inversion implemented in the VBA toolbox^90^, with condition-specific inequity aversion parameters estimated jointly within each participant-level model. Thus, fitting the model to the choice data obtained in different blocks of the altruism task yielded participant-specific estimates of advantageous and disadvantageous inequity aversion (*α* and *β*) for each social context (task condition).

To test whether fairness preferences varied across social contexts (i.e., task conditions differing in partner characteristics), we conducted Friedman tests separately for *α* and *β*, followed by post-hoc pairwise comparisons as described above. In addition, model-derived parameters for inequity aversion were entered into a mixed linear model to examine their association with common patterns of ongoing thought (see below). Because *α* and *β* are estimated latent variables, these analyses necessarily inherit uncertainty from the parameter estimation procedure. Accordingly, these associations should be interpreted cautiously as relationships between estimated fairness preferences and ongoing thought patterns rather than direct observations of latent preferences themselves.

***Characterizing common patterns of ongoing thought (MDES)***. Thought patterns were identified using principal component analysis (PCA) with Varimax rotation following previous approaches (*ThoughtSpace* GitHub repository^91^). The PCA was implemented in Python (version 3.8.16) using the *Scikit-learn*^92^ and *Scipy* packages^93^. Before applying PCA, MDES data were z-scored using *Scipy’s z-score* function. Varimax rotation was employed to enhance the interpretability of the solution while preserving their orthogonality, consistent with standard practices of analysing MDES data^6,24,26,27,51^. The PCA was conducted on the joint (online and in-person) MDES data of all participants and observations (14 items; Table 1). To assess the suitability of the MDES data for PCA, we used the Kaiser-Meyer-Olkin (KMO) measure of sampling adequacy and Bartlett’s test of sphericity. The four components with eigenvalues greater than 1 were retained as dependent variables for the linear mixed models that examined the link between thought patterns and generosity, social context (task conditions: partner characteristics), and task environment (online, in-person). Loadings of components 2 and 4 were reversed for easier interpretation. Note that thought patterns were based solely on MDES data of the altruism task, excluding the MDES data of the free viewing task obtained for the in-person sample (the latter were used to test if identified thought patterns generalize to non-altruism related settings in a separate robustness check).

***Reliability***,*** stability***,*** and generalizability of thought patterns.*** We conducted several reliability and robustness checks on the identified thought patterns. First, we conducted a bootstrapped split-half reliability analysis to assess the robustness of the PCA solution for subsets of our MDES data using the *RHom* module in the *ThoughtSpace* package, following previous implementations^27,29,53^. In this analysis, the MDES data were repeatedly split into two random halves, components were computed separately for each half, and the similarity between corresponding components was evaluated using the R-homologue score. Second, we verified the stability of the components over time by exploring their intraclass correlation (ICC) across the MDES assessments in the altruism task, separately for each social context (task condition) and task environment (online, in-person). The ICC was computed using a two-way mixed-effects model with consistency and mean measures (k = 3) as recommended by^94^ (for guidelines see^95^). Third, to confirm that the thought patterns were present in both samples (task environments: online, in-person) individually, we performed separate PCAs (with four components specified for extraction) on MDES data from each sample (task environment). We then computed Pearson correlations of each participant’s estimated, corresponding PCA component scores between the individual and joint sample analyses (note that component order may differ between analyses). Fourth, we used the projection method^29,44^ to further evaluate the consistency of identified thought patterns across samples (task environments: online, in-person). Specifically, we projected the four identified thought patterns in the in-person sample onto MDES data from the online sample. Projection involved calculating the dot product between the component loadings from the in-person sample and the z-scored MDES data from the online sample. Fifth, we tested for the generalizability of thought patterns beyond altruistic choice settings to task settings that did not require meaningful social actions. To this end, we projected thought patterns identified in the altruism task onto MDES data observed during naturalistic viewing of complex daily scenes (free viewing task data obtained on a separate day from the altruism task, averaged delay: 7 days). Here, projection involved calculating the dot product between the PCA-based component loadings from the altruism task and the z-scored MDES items from the free viewing task.

***Association between common thought patterns and generosity***,*** social context***,*** and task environment***. We next addressed our key question: Do common patterns of ongoing thoughts reflect changes in social and contextual features of the choice setting? Specifically, we tested whether thought patterns systematically varied as a function of generosity, social context, and task environment. To this end, we estimated four linear mixed models, one for each thought pattern *X* identified in the PCA (Fig. 2a). Predictor variables included: *(1)* generosity level (high vs. low, based on a median split of block-wise generosity scores due to bivariate data distributions; Fig. 1b), *(2)* social context (task condition: identifiable distressed, identifiable neutral, unidentifiable interaction partner), and *(3)* task environment (online vs. in-person). To control for nuisance variation, we included age and mean-centered generosity within each generosity group as covariates (see^26^ for prior implementations; see also^96,97^ for evidence of age effects on prosocial behavior), along with random intercepts for participants. The model did not include interaction terms based on the results of a formal model comparison that favored the simpler model (Supplementary Table S2). The model specification was:

2 $$\begin{aligned}\:Xij = \beta_0+\beta_1\:Generosity_{ij} +\beta_2\:Social\:Context_{ij} +\beta_3\:Environment_j +\beta_4\:Age_j \\+ \beta_5\:(Generosity_{ij}\:-\:mean\:Generosity\:within\:group_j)\:+\:u_j +\varepsilon_{ij}\end{aligned}$$

where *i* indexes observations and *j* indexes participants; *u*_*j*_ represents a participant-specific random intercept (u_j_ ∼ N(0,σ_u_^2^)), and *ε*_*ij*_ represents the residual error term (ε_ij_ ∼ N(0,σ^2^)).

A total of 320 participants (joint sample, 1,920 MDES observations) contributed data. Like previous implementations of this approach^26^, models were fitted with restricted maximum-likelihood estimation using the *lme4* package in R (version 4.2.3)^98^. P-values were obtained with *lmerTest*^99^ in R and Bonferroni corrected. The Satterthwaite approximation was used to calculate the degrees of freedom. Contrasts were set to *contr.sum*, such that the model intercept represented the grand mean across all conditions, and estimated coefficients for factors with two levels reflected half the difference between conditions^100^. The *emmeans* package was employed to estimate marginal means for visualizing the effects of generosity, social context, and task environment (Fig. 3)^101^.

***Time-Lagged Analyses of Thought and Behavior.*** For exploratory purposes, supplemental models examined the temporal relationship between ongoing thought pattern 4 (ruminative social cognition) and social behavior (generosity). Two complementary sets of time-lagged mixed-effects analyses were conducted to examine whether variation in one variable was temporally associated with variation in another across adjacent trials, providing insight into temporal ordering—necessary, though not sufficient, for causal inference. These two supplementary time-lagged analyses focused on thought pattern 4, which exhibited the most robust association with generosity (Table 3).

*Temporal association between thought pattern 4 and subsequent generosity.* We first fit a binomial mixed-effects model to test whether ruminative social cognition was associated with generosity on the *subsequent* trial. Generosity was coded as a binary outcome (0 = selfish, 1 = generous) for the trial immediately following each MDES thought probe. Fixed effects included thought pattern 4 (ruminative social cognition), participants’ task environment (online vs. in-person), and age (mean-centered). A random intercept for participant accounted for repeated measures. We excluded MDES-following trials without a self–other trade-off (as the trade-off is crucial for our definition of trial-wise generosity), yielding 1,345 observations. Logistic mixed-effects models were estimated using the glmer function (lme4 package, R) with Wald z tests for significance.

*Temporal association between preceding generosity and thought pattern 4.* To examine associations in the reverse temporal direction, a linear mixed-effects model tested whether generosity on the trial immediately *preceding* each MDES probe was associated with thought pattern 4 (ruminative social cognition). Fixed effects included MDES probe-preceding generosity (0 = selfish, 1 = generous), participants’ task environment and age, with a random intercept for participant. By-subject random slopes for preceding generosity were included to account for individual differences in the carryover effect. Degrees of freedom and p-values were estimated using Satterthwaite’s approximation (*lmerTest* package, R). We excluded thought probe-preceding generosity trials without a self–other trade-off, yielding 1,535 observations.

***Association between thought patterns and social fairness preferences.*** Finally, we examined whether individual differences in fairness preferences —known drivers of generosity^89^—were associated with common patterns of ongoing thought. Here, we implemented a modified version of Eq. 2 in which the predictor generosity was replaced with model-based estimates of fairness preferences derived from the Fehr–Schmidt framework. For each parameter (*α* and *β*), participants were split into high vs. low groups via median splits, due to the bivariate distribution of fairness estimates (Supplementary Fig. S3). This supplemental model was restricted to thought pattern 4, the only pattern that showed a robust association with generosity after Bonferroni correction in our main analysis. This approach examined whether fairness preferences were related to individual differences in thought content associated with generosity in altruistic choice settings.

## Supplementary Information

Below is the link to the electronic supplementary material.

Supplementary Material 1

## Data Availability

The datasets analysed during the current study are deposited on the Open Science Framework and available at: https://doi.org/10.17605/OSF.IO/MV6NR.

## Code availability

MDES thought data were processed using the open source “ThoughtSpace” GitHub repository^91^. Source code underlying the linear mixed model analysis is openly available here^26^.
