# Supplementary material for: Common thought patterns reflect generosity, fairness, and social context
Source: Sci Rep. 2026 Jul 31;16:23697. doi: 10.1038/s41598-026-61078-5 (PMC13427752; doi:10.1038/s41598-026-61078-5)
Supplement: Supplementary file 1 — Supplementary Material 1 [file 41598_2026_61078_MOESM1_ESM.pdf]

Supplemental Information (SI)

Common Thought Patterns Reflect Generosity, Fairness, and Social Context

Lisa M. Bas [1,2,3*], Ruien Wang [1], Jonathan Smallwood [1], and Anita Tusche [1,4*]

[1] Queen's University, Department of Psychology, Kingston, K7L 3L3, Canada

[2] Department of Psychology, Julius Maximilians University of Würzburg, 97070 Würzburg, Germany

[3] Department of Child and Adolescent Psychiatry, Psychosomatics and Psychotherapy, University Hospital, Julius Maximilians University of Würzburg, 97080 Würzburg, Germany

[4] Queen's University, Centre for Neuroscience Studies, Kingston, K7L 3L3, Canada

* Corresponding authors:

Dr. Lisa M. Bas; lisa.bas@queensu.ca

Dr. Anita Tusche; anita.tusche@queensu.ca

Queen's University, 99 University Ave, Kingston, ON K7L 3N6

Table of Contents

Supplementary Figure S1. Individual differences in mean generosity across conditions (identifiable distressed, identifiable neutral, unidentifiable). 3

Supplementary Figure S2. Correlation between estimated parameters disadvantageous inequity (α) and advantageous inequity (β) for the joint sample 4

Supplementary Figure S3. Distribution of model-based estimates of participants' advantageous inequity aversion (β) and disadvantageous inequity aversion (α), separately for each condition (social context) of the altruism task. 5

Supplemental Figure S4. Scree plot from separate principal component analysis (PCA). 6

Supplementary Table S1. Reliability scores (intraclass-correlations, ICC) for each principal component within task conditions, tasks, and online and in-person samples for the altruism task and the free viewing task. 7

Supplementary Table S2. Model comparison (Equation 2) for each distinct component. 8

Supplementary Table S3. Ongoing thoughts are sensitive to people's generosity, environment, and social context 9

Supplementary Table S4. Common thought patterns predict individuals' generosity level (high/low). 10

Supplementary Table S5. Common thought patterns predict continuous generosity scores. 11

Supplementary Table S6. Thought pattern 4 (ruminative social cognition) predicts continuous generosity scores, when context is removed. 12

Supplementary Table S7. Ruminative social cognition (thought pattern 4) is associated with increased generosity levels (0/1) on trials immediately following MDES thought probes. 13

Supplementary Table S8. Generosity (0/1) on trials immediately preceding MDES thought probes is associated with more ruminative social cognition (thought pattern 4). 14

Supplementary Table S9. Continuous estimates of advantageous inequity aversion predict the expression of a common thought pattern (ruminative social cognition) 15

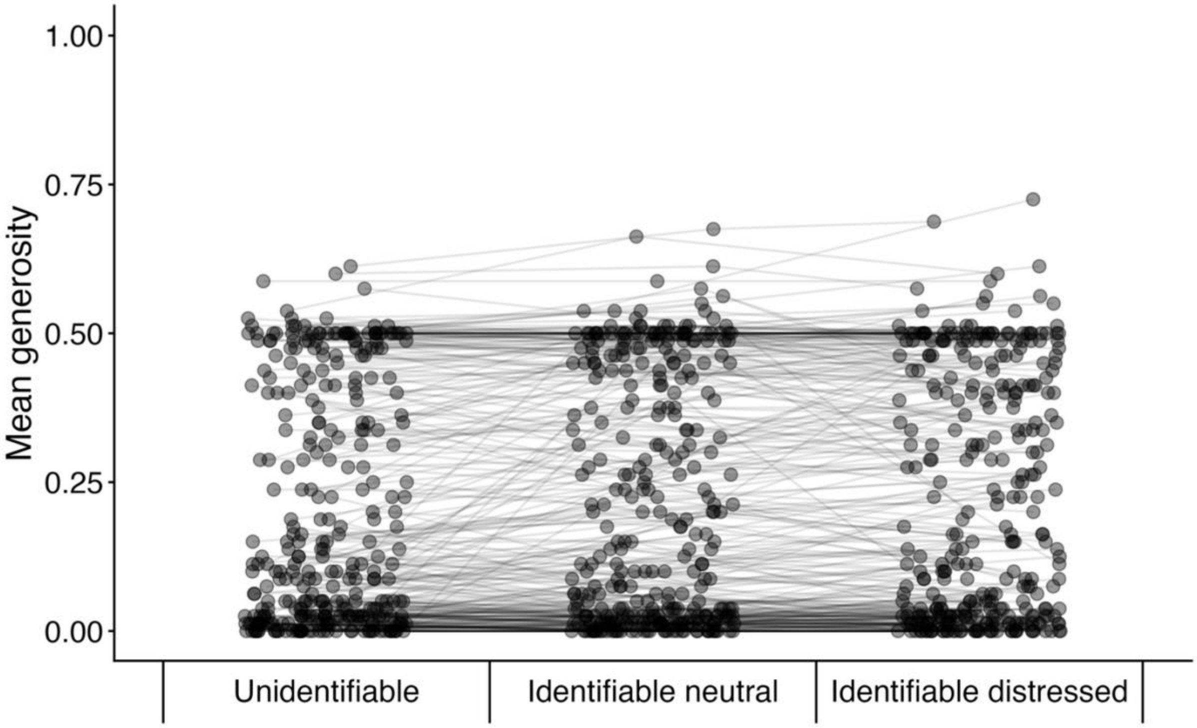

Supplementary Figure S1. Individual differences in mean generosity across conditions (identifiable distressed, identifiable neutral, unidentifiable). Black data points and lines indicate individual differences across participants (n = 320).

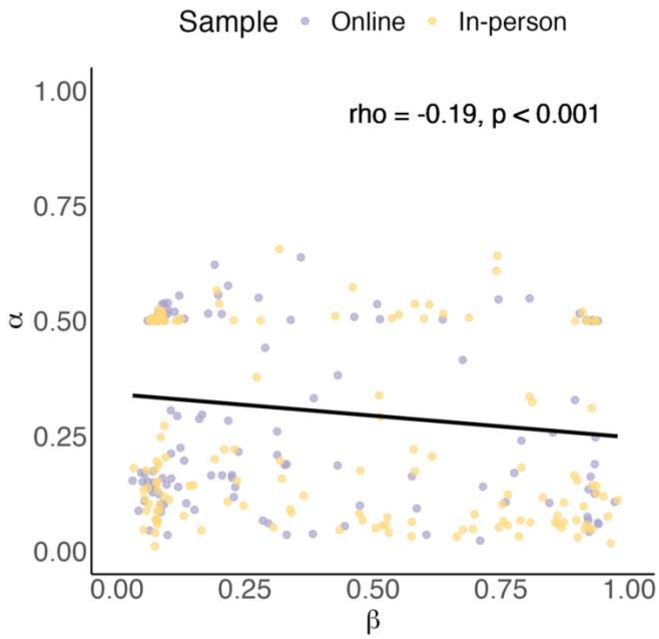

Supplementary Figure S2. Correlation between estimated parameters disadvantageous inequity (α) and advantageous inequity (β) for the joint sample. The black line indicates the linear trend line. Sample-specific data points (task environment) are visualized in purple (online) and yellow (in-person).

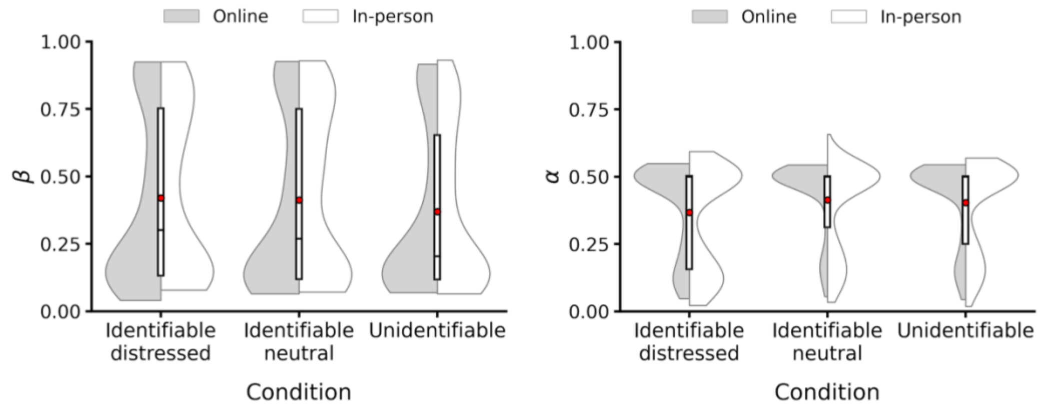

Supplementary Figure S3. Distribution of model-based estimates of participants' advantageous inequity aversion (β) and disadvantageous inequity aversion (α), separately for each condition (social context) of the altruism task.

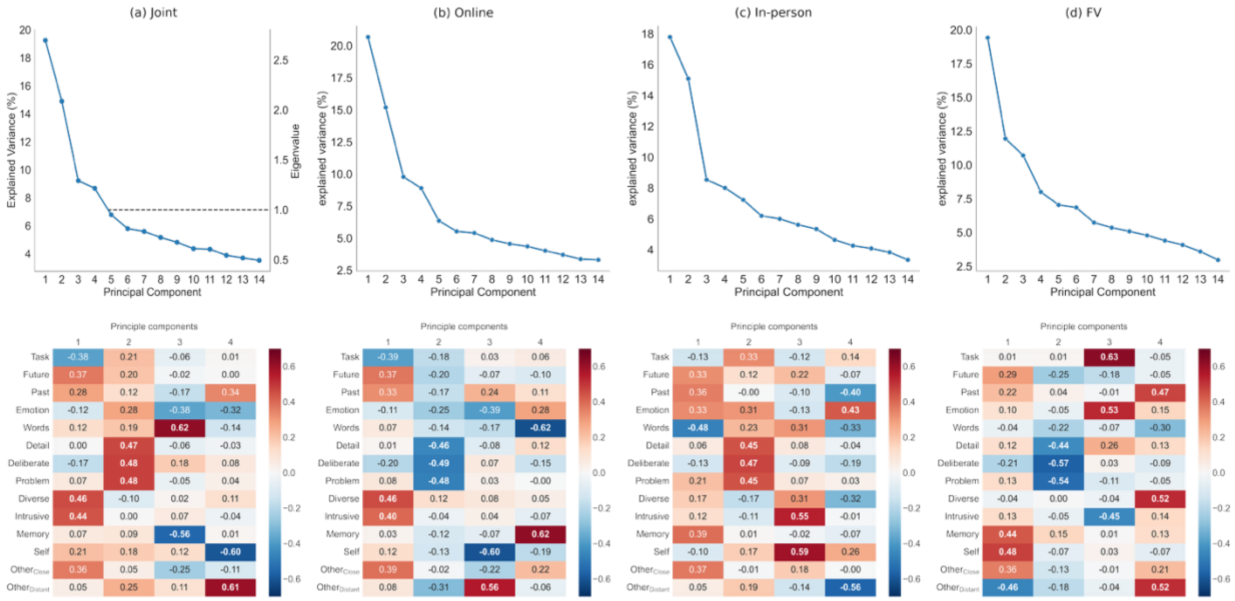

Supplemental Figure S4. Scree plot from separate principal component analysis (PCA). PCA applied to the thought MDES data from the altruism task from (a) the joint sample (online and in-person), (b) the online sample, (c) the in-person sample, (d) the separate free viewing (FV) task to identify common patterns of thought (x-axis = component number, left y-axis = % variance explained, and right y-axis = eigenvalues [displayed for the joint sample]). Below each scree plot is the Varimax-rotated component matrix from the PCA of the MDES thought data (14 items; see Table 1) for each specific sample and task (note that component ordering may differ between the joint and sample-specific PCAs). Values greater than or equal to 0.4 and less than or equal to -0.4 are in bold.

Supplementary Table S1. Reliability scores (intraclass-correlations, ICC) for each principal component within task conditions, tasks, and online and in-person samples for the altruism task and the free viewing task.

Task	Sample (Environment)	Condition (Social context)	Component 1	Component 2	Component 3	Component 4
Altruism Task	Online	Identifiable distressed	0.82	0.89	0.73	0.84
		Identifiable neutral	0.81	0.80	0.75	0.81
		Unidentifiable (control)	0.78	0.81	0.74	0.80
Altruism Task	In-person	Identifiable distressed	0.75	0.70	0.74	0.68
		Identifiable neutral	0.73	0.68	0.76	0.68
		Unidentifiable (control)	0.76	0.73	0.68	0.68
Free Viewing Task	In-person	-	0.79	0.78	0.76	0.71

Note. All correlations reached significance (all p’s < 0.001).

Supplementary Table S2. Model comparison (Equation 2) for each distinct component.

	Component 1		Component 2		Component 3		Component 4	
	M1	M2	M1	M2	M1	M2	M1	M2
AIC	6040.1	6047.2	5765.9	5770.4	5001.8	5007.5	5121.0	5111.9
BIC	6090.2	6136.1	5816.0	5859.3	5051.8	5096.5	5171.1	5200.9
#parameters	9	16	9	16	9	16	9	16

Note. M1 = Model 1 without interaction effects (Eq. 2), M2 = Model 2 including two-way interactions between factors of interest (task environment, generosity, and social context), AIC = Akaike Information Criterion (lower values indicate a better balance between model fit and complexity), BIC = Bayesian Information Criterion (lower values indicate a better model but penalizes additional parameters more than AIC); 15 out of the 16 values favor the simpler Model 1 that balances fit and interpretability.

Supplementary Table S3. Ongoing thoughts are sensitive to people's generosity, environment, and social context (models match those reported in Table 3, but use the continuous number of generous choices per task block instead of binarized high/low generosity as a predictor variable).

	Thought Pattern 1 (Off-task episodic social cognition)				Thought Pattern 2 (Detailed task focus)				Thought Pattern 3 (Negative dialogue)				Thought Pattern 4 (Ruminative social cognition)			
Predictors	*Estimates*	*CI*	*Statistic*	*p*	*Estimates*	*CI*	*Statistic*	*p*	*Estimates*	*CI*	*Statistic*	*p*	*Estimates*	*CI*	*Statistic*	*p*
(Intercept)	0.85	[0.30, 1.40]	3.04	0.002‡	-0.28	[-0.83, 0.26]	-1.02	0.308	0.12	[-0.30, 0.53]	0.56	0.575	-0.11	[-0.48, 0.27]	-0.56	0.575
Environment (Online)	-0.27	[-0.43, -0.11]	-3.3	0.001‡	-0.03	[-0.19, 0.13]	-0.35	0.723	-0.21	[-0.33, -0.09]	-3.45	0.001‡	-0.01	[-0.11, 0.10]	-0.1	0.918
Generosity (Continuous)	0.31	[-0.12, 0.75]	1.41	0.158	0.45	[0.04, 0.86]	2.14	0.032	-0.34	[-0.67, -0.01]	-2.02	0.044	0.68	[0.36, 1.01]	4.11	<0.001‡
Context (Identifiable distressed)	0.11	[0.05, 0.17]	3.63	<0.001‡	-0.08	[-0.14, -0.02]	-2.76	0.006‡	0	[-0.05, 0.04]	-0.08	0.939	0.18	[0.13, 0.23]	7.01	<0.001‡
Context (Identifiable neutral)	-0.05	[-0.11, 0.01]	-1.7	0.089	0.01	[-0.05, 0.06]	0.3	0.764	-0.07	[-0.12, -0.02]	-2.98	0.003‡	0.13	[0.08, 0.18]	5.26	<0.001‡
Age	-0.03	[-0.05, -0.01]	-3.39	0.001‡	0.01	[-0.01, 0.03]	0.7	0.485	0	[-0.02, 0.01]	-0.2	0.844	0	[-0.01, 0.01]	-0.22	0.826

Note. ‡ indicates p-values that survive Bonferroni correction for multiple comparisons across the four independent models. CI = confidence interval.

Supplementary Table S4. Common thought patterns predict individuals' generosity level (high/low).

Predictors	*Estimates*	*Std. Error*	*Statistic (z)*	*P uncorrected*
(Intercept)	-0.12	0.37	-0.32	0.747
Thought pattern 1	0.03	0.10	0.31	0.760
Thought pattern 2	0.16	0.11	1.44	0.149
Thought pattern 3	-0.28	0.13	-2.05	0.040
Thought pattern 4	0.36	0.13	2.86	0.004
Environment (online)	-0.92	0.39	-2.34	0.019
Context (identifiable distressed)	0.28	0.13	2.12	0.034
Context (identifiable neutral)	0.23	0.13	1.81	0.070

Note. The robust associations between thought patterns and social features of the choice setting (reported in the main text) raise the possibility that ongoing thoughts may also predict meaningful social behavior, such as generosity. To test this, we reversed the direction of the regression and modeled generosity level (high vs. low) as the dependent variable, with the four thought patterns (components) entered as predictors. The model also included task environment (online vs. in-person), social context (partner characteristics), and random intercepts for participants, mirroring the structure of the main analyses. Consistent with the main-text findings, two thought patterns were associated with variability in generosity (uncorrected p-values). Internal negative dialogue (thought pattern 3) was associated with *reduced* generosity ($\beta = -0.28$, SE = 0.13, z = -2.05, $p < 0.05$), whereas ruminative social cognition (thought pattern 4) was associated with *greater* generosity ($\beta = 0.36$, SE = 0.13, z = 2.86, $p < 0.01$). Thought patterns 1 and 2 were not significantly linked with generosity levels (p's > 0.149). Replicating patterns reported in the main text, social context was again associated with increased generosity: participants were more generous toward an identifiable distressed partner ($\beta = 0.28$, SE = 0.13, z = 2.12, $p < 0.05$). In addition, generosity was lower in the online environment relative to in-person testing ($\beta = -0.92$, SE = 0.39, z = -2.34, p = 0.019). Age was removed from this model to enable model convergence.

For completeness, we also examined whether thought patterns could predict task environment or social context. These supplemental models matched the structure above but used different dependent variables. In Supplemental Model 2 (task environment as the outcome), no thought pattern was significantly associated with task environment (all p's > 0.259). In Supplemental Model 3 (social context as the outcome), ruminative social cognition (thought pattern 4) was significantly associated with social context ($p < 0.001$), whereas all other components were not (p's > 0.065). Generosity level was not included in these two supplemental models to permit convergence. Together, these supplemental analyses demonstrate that distinct patterns of ongoing thought systematically relate to differences in generosity and, to a lesser extent, to features of the social interaction context.

Supplementary Table S5. Common thought patterns predict continuous generosity scores.

Predictors	*Estimates*	*Std. Error*	*df*	*Statistic (t)*	$P_{uncorrected}$
(Intercept)	18.64	4.39	316	4.243	< 0.001
Thought pattern 1	0.09	0.14	1675	0.657	0.511
Thought pattern 2	0.28	0.15	1686	1.863	0.063
Thought pattern 3	-0.60	0.19	1666	-3.208	0.001
Thought pattern 4	0.33	0.17	1649	1.886	0.059
Context (identifiable distressed)	0.88	0.18	1596	4.923	< 0.001
Context (identifiable neutral)	0.57	0.17	1595	3.199	0.001
Environment (online)	-2.39	1.29	316	0.704	0.482
Age	0.11	0.16	317	-1.860	0.064

Note. To improve interpretability, generosity scores were multiplied by 100 to reflect the percentage of generous choices. This transformation linearly rescales estimates and standard errors but does not change the significance or direction of effects. See Supplementary Table S6 for estimates of thought patterns 4 when context is removed from the model.

Supplementary Table S6. Thought pattern 4 (ruminative social cognition) predicts continuous generosity scores, when context is removed.

Predictors	*Estimates*	*Std. Error*	*df*	*Statistic (t)*	$P_{uncorrected}$
(Intercept)	18.60	4.37	316	4.253	< 0.001
Thought pattern 1	0.12	0.15	1681	0.852	0.394
Thought pattern 2	0.22	0.16	1693	1.448	0.148
Thought pattern 3	-0.68	0.19	1671	-3.629	< 0.001
Thought pattern 4	0.74	0.17	1649	4.376	< 0.001
Environment (online)	-2.39	1.28	317	-1.871	0.062
Age	0.11	0.16	316	0.717	0.474

Note. To improve interpretability, generosity scores were multiplied by 100 to reflect the percentage of generous choices. This transformation linearly rescales estimates and standard errors but does not change the significance or direction of effects.

Supplementary Table S7. Ruminative social cognition (thought pattern 4) is associated with increased generosity levels (0/1) on trials immediately following MDES thought probes.

Predictors	*Estimates*	*Std. Error*	*Statistic (z)*	*P*
(Intercept)	-1.84	0.14	-13.28	<.001
Thought pattern 4	0.18	0.08	2.11	0.035
Environment (online)	0.18	0.13	1.36	0.171
Age	0.01	0.02	0.44	0.662

Note. This supplementary time-lagged analysis focused on thought pattern 4, which exhibited the most robust association with generosity (Table 3) and with subsequent social behavior (i.e., generosity on the following trial). Accordingly, we implemented a reduced model targeting this focal thought component. Including all thought patterns in the model does not qualitatively alter the results. Social context (task condition) was excluded from the model because it is fixed across multiple trials and therefore represents a coarse block-level factor, whereas the present model is intended to isolate trial-level dynamics.

Supplementary Table S8. Generosity (0/1) on trials immediately preceding MDES thought probes is associated with more ruminative social cognition (thought pattern 4).

Predictors	Estimates	Std. Error	df	Statistic (t)	P
(Intercept)	-0.00	0.05	312	-0.039	0.968
Generosity on trial before MDES probe	0.13	0.06	1308	2.166	0.031
Environment (online)	-0.02	0.06	302	-0.316	0.752
Age	-0.01	0.01	293	-0.801	0.424

Note. This supplementary time-lagged analysis focused on thought pattern 4, which exhibited the most robust association with generosity (Table 3). Social context (task condition) was excluded from the model because it is fixed across multiple trials and therefore represents a coarse block-level factor, whereas the present model is intended to isolate trial-level dynamics.

Supplementary Table S9. Continuous estimates of advantageous inequity aversion predict the expression of a common thought pattern (ruminative social cognition)

Predictors	*Estimates*	*Std. Error*	*df*	*Statistic (t)*	$P_{uncorrected}$
(Intercept)	-0.11	0.20	409	-0.566	0.572
β (advantageous inequity aversion)	0.57	0.13	1031	4.580	< 0.001
α (disadvantageous inequity aversion)	-0.13	0.16	1769	-0.840	0.401
Environment (online)	-0.01	0.05	314	-0.085	0.932
Context (identifiable distressed)	0.17	0.03	1613	6.641	< 0.001
Context (identifiable neutral)	0.14	0.03	1594	5.351	< 0.001
Age	-0.00	0.01	313	-0.344	0.731
